# Supplementary material for: IFN-I Score and Rare Genetic Variants in Children with Systemic Lupus Erythematosus
Source: Biomedicines. 2024 Jun 3;12(6):1244. doi: 10.3390/biomedicines12061244 (PMC11200921; doi:10.3390/biomedicines12061244)
Supplement: Supplementary file 1 [file biomedicines-12-01244-s001.zip › biomedicines-2969400-supplementary.docx]

Supplementary Table S1. Correlations between IFN-I score and SLE parameters.

| **Parameter** | **IFN-I score** | | **IFI44** | | **IFI44L** | | **IFIT3** | | **MX1** | |
| --- | --- | --- | --- | --- | --- | --- | --- | --- | --- | --- |
|  | **r** | **p** | **r** | **p** | **r** | **p** | **r** | **p** | **r** | **p** |
| Leucopenia | 0.420 | 0.023 | 0.509 | 0.005 |  |  |  |  | 0.392 | 0.036 |
| Lymphopenia | 0.399 | 0.032 | 0.423 | 0.022 |  |  |  |  | 0.434 | 0.019 |
| Pericarditis |  |  | 0.999 | 0.018 | 0.999 | 0.011 |  |  |  |  |
| Raynaud’s phenomenon | 0.470 | 0.010 | 0.651 | 0.0001 |  |  |  |  |  |  |
| Livedoid rash | 0.601 | 0.001 | 0.926 | 0.0001 |  |  | 0.437 | 0.018 |  |  |
| Fever |  |  |  |  |  |  |  |  | 0.428 | 0.011 |
